# Supplementary material for: Large-Scale Application of Double-Stranded RNA Shows Potential for Reduction of Sacbrood Virus Disease in Apis cerana Apiaries
Source: Viruses. 2023 Mar 31;15(4):897. doi: 10.3390/v15040897 (PMC10145737; doi:10.3390/v15040897)
Supplement: Supplementary file 1 [file viruses-15-00897-s001.zip › viruses-2183046-supplementary.pdf]

Supplementary Materials

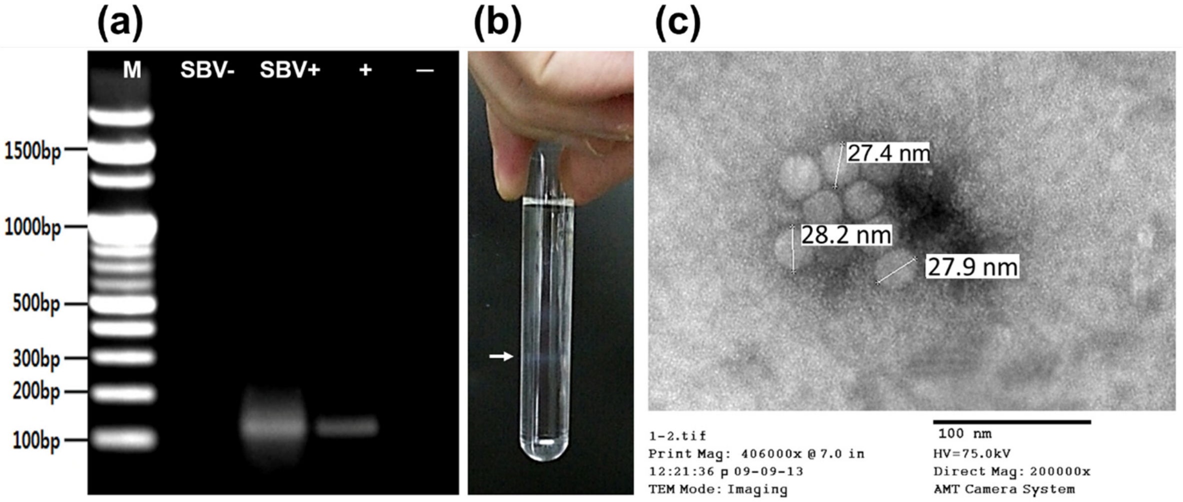

Figure S1. Detection and isolation of sacbrood virus (SBV) from infected honeybee larvae. (**a**) SBV in infected *Apis cerana* larvae was detected with RT-qPCR and confirmed by electrophoresis, with an expected band of 131 bp in length. SBV+ and SBV- are samples with positive and negative detection, respectively. "+" and "-" are the positive and negative controls with SBV recombinant DNA and without a DNA template, respectively. (**b**) A band of purified SBV from infected larvae in 40% sucrose gradient density (indicated by the arrow). (**c**) Viral particles of SBV, with a size of 27.8 ± 0.4 nm and a shape similar to that of other picornaviruses, were observed and measured under transmission electron microscopy.

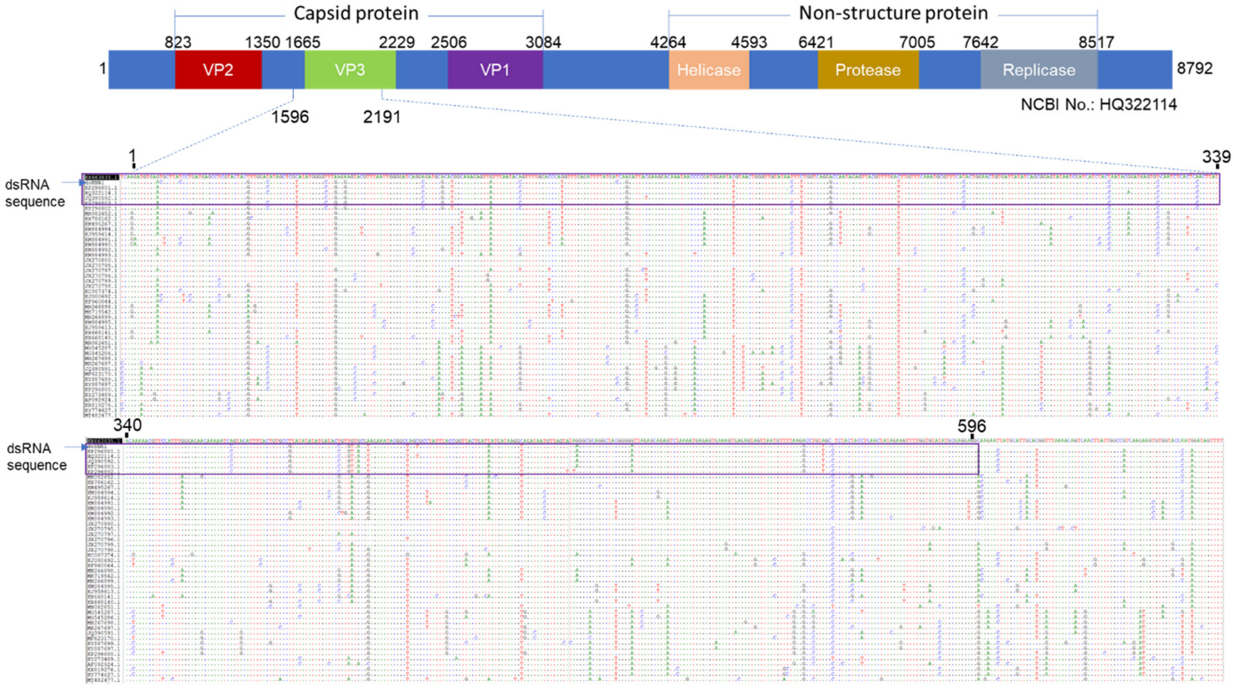

Figure S2. Alignment of dsRNA sequences with SBV genome sequences. The complete genomes of SBV from NCBI were used for alignment; NCBI accession numbers are shown. The position (596 bp) with the highest sequence similarity of SBV detected in the Republic of Korea was selected as the target of RNA interference. The target position (596 bp) is marked in the purple boxes. The dsRNA sequence produced in this study is indicated by the arrows.

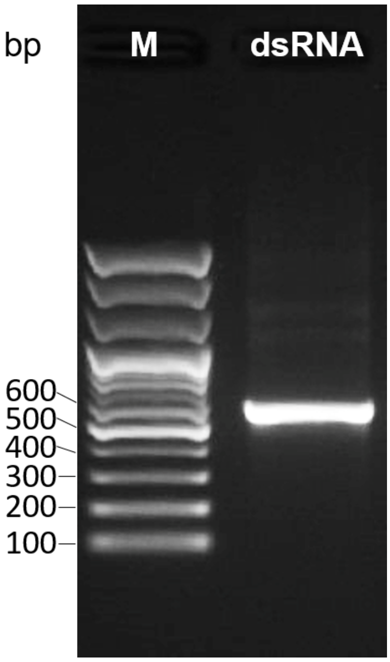

Figure S3. Double-stranded RNA (dsRNA) of sacbrood virus VP3. The dsRNA (185 ng) was loaded into a 1% agarose gel for electrophoresis. The 596 bp band corresponds to dsRNA. “M” is a 100 kb DNA marker.

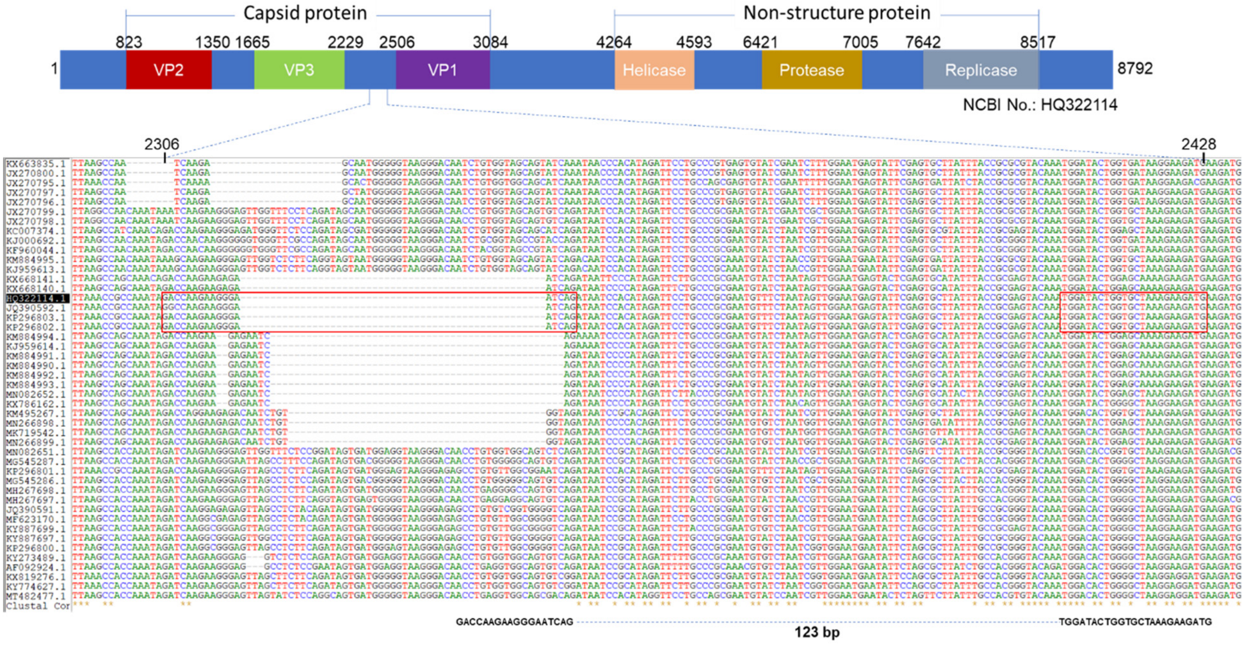

Figure S4. Alignment of SBV DNA sequences for specific primer design. Complete genomes of SBV collected from NCBI were used for alignment to design primers for specific detection of the SBV genotype that causes disease in *Apis cerana* in the Republic of Korea. A forward primer was designed for the specific mutation position of the Korean SBV genotype. The positions of forward and reverse primers are denoted with the red boxes. A fragment 123 bp long was amplified using the primer pair. The positions of different genes on the genome of SBV and the NCBI accession numbers of the sequences are shown.

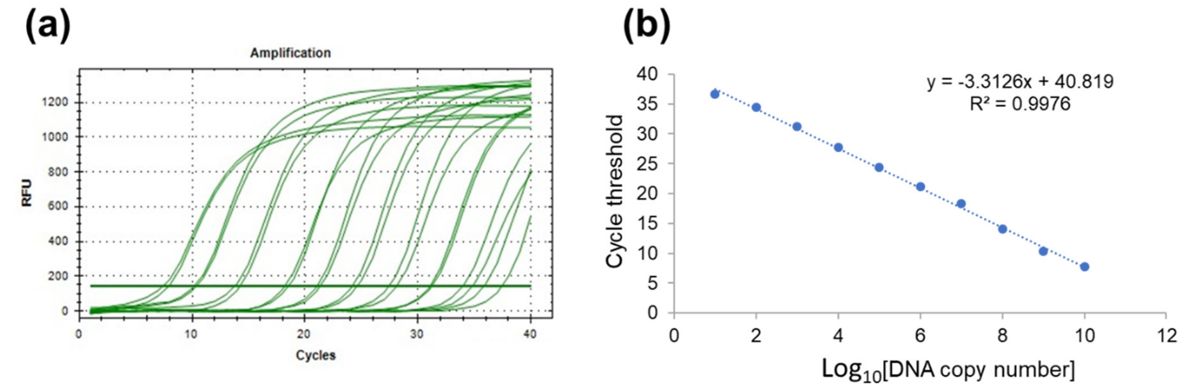

Figure S5. Standard curves of sacbrood virus (SBV) DNA amplification. (**a**) A 10-fold serial dilution of SBV recombinant DNA, namely, from 10^{10} to 10^{1} copies, was used for triplicate PCRs. (**b**) The correlation between the initial number of DNA copies (log_{10}-transformed values) and the cycle threshold of amplification is shown.

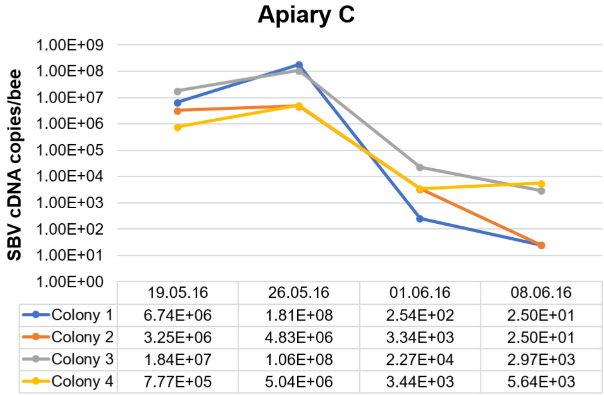

	19.05.16	26.05.16	01.06.16	08.06.16
Colony 1	6.74E+06	1.81E+08	2.54E+02	2.50E+01
Colony 2	3.25E+06	4.83E+06	3.34E+03	2.50E+01
Colony 3	1.84E+07	1.06E+08	2.27E+04	2.97E+03
Colony 4	7.77E+05	5.04E+06	3.44E+03	5.64E+03

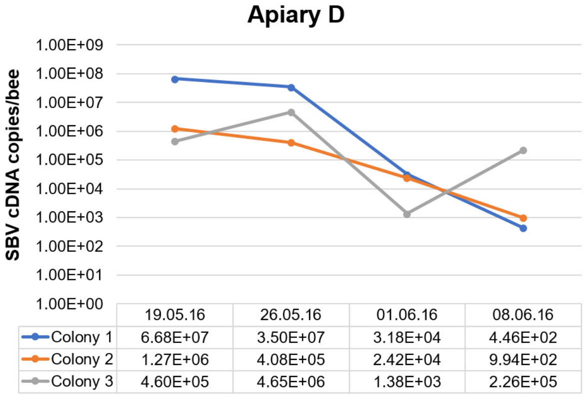

	19.05.16	26.05.16	01.06.16	08.06.16
Colony 1	6.68E+07	3.50E+07	3.18E+04	4.46E+02
Colony 2	1.27E+06	4.08E+05	2.42E+04	9.94E+02
Colony 3	4.60E+05	4.65E+06	1.38E+03	2.26E+05

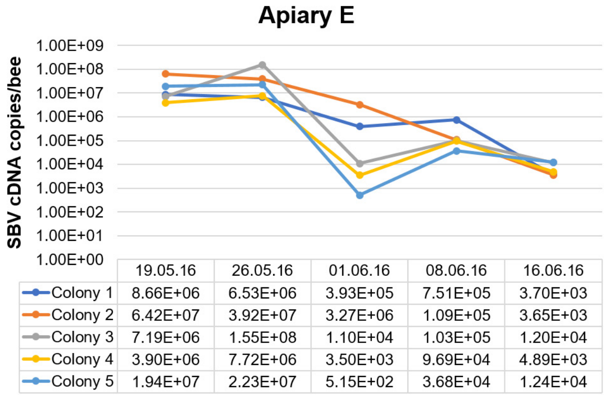

	19.05.16	26.05.16	01.06.16	08.06.16	16.06.16
Colony 1	8.66E+06	6.53E+06	3.93E+05	7.51E+05	3.70E+03
Colony 2	6.42E+07	3.92E+07	3.27E+06	1.09E+05	3.65E+03
Colony 3	7.19E+06	1.55E+08	1.10E+04	1.03E+05	1.20E+04
Colony 4	3.90E+06	7.72E+06	3.50E+03	9.69E+04	4.89E+03
Colony 5	1.94E+07	2.23E+07	5.15E+02	3.68E+04	1.24E+04

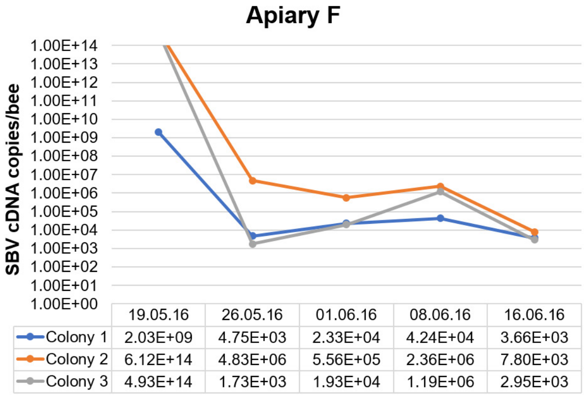

	19.05.16	26.05.16	01.06.16	08.06.16	16.06.16
Colony 1	2.03E+09	4.75E+03	2.33E+04	4.24E+04	3.66E+03
Colony 2	6.12E+14	4.83E+06	5.56E+05	2.36E+06	7.80E+03
Colony 3	4.93E+14	1.73E+03	1.93E+04	1.19E+06	2.95E+03

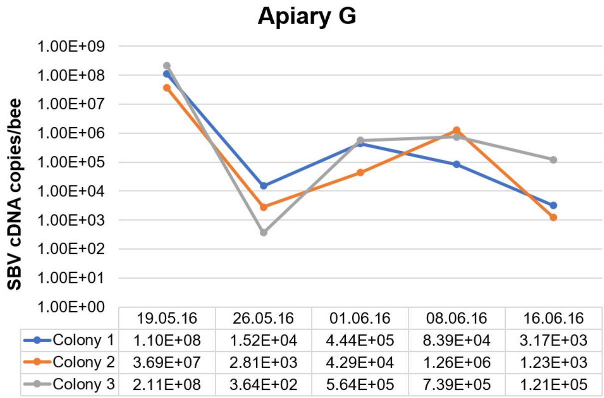

	19.05.16	26.05.16	01.06.16	08.06.16	16.06.16
Colony 1	1.10E+08	1.52E+04	4.44E+05	8.39E+04	3.17E+03
Colony 2	3.69E+07	2.81E+03	4.29E+04	1.26E+06	1.23E+03
Colony 3	2.11E+08	3.64E+02	5.64E+05	7.39E+05	1.21E+05

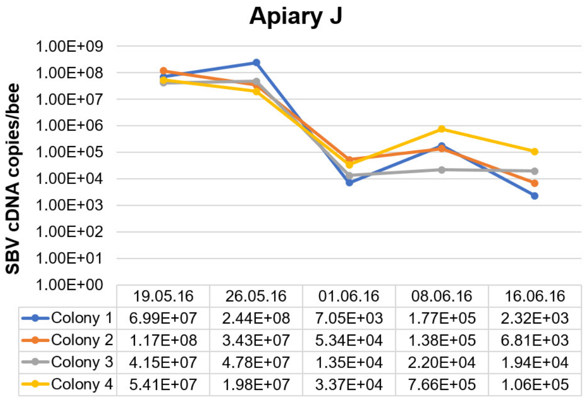

	19.05.16	26.05.16	01.06.16	08.06.16	16.06.16
Colony 1	6.99E+07	2.44E+08	7.05E+03	1.77E+05	2.32E+03
Colony 2	1.17E+08	3.43E+07	5.34E+04	1.38E+05	6.81E+03
Colony 3	4.15E+07	4.78E+07	1.35E+04	2.20E+04	1.94E+04
Colony 4	5.41E+07	1.98E+07	3.37E+04	7.66E+05	1.06E+05

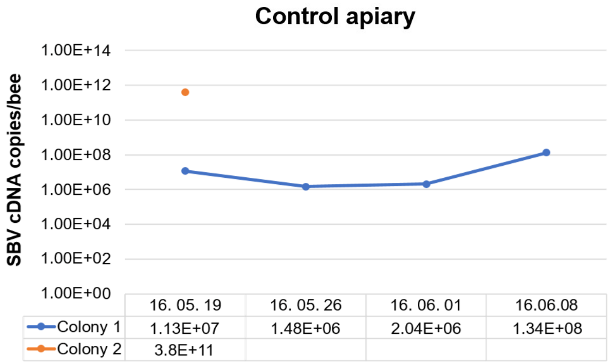

	16. 05. 19	16. 05. 26	16. 06. 01	16.06.08
Colony 1	1.13E+07	1.48E+06	2.04E+06	1.34E+08
Colony 2	3.8E+11			

Figure S6. Quantitative detection of sacbrood virus (SBV) during the dsRNA treatment period. Quantitative detection of SBV was done before dsRNA administration and was continued after dsRNA administration with a 1-week interval. A total of 22 colonies from six apiaries (C-G and J) were selected for the RT-qPCR test. Two colonies from different apiaries with no treatment of dsRNA were also selected for detection of SBV. The colony 2 in the control group

collapsed after one week. Therefore, only one test was done for this colony with SBV DNA, which was 3.8×10^{11} copies/bee. The dsRNA treatment and SBV detection were done from May 19th to June 16th, 2016. The date of each test is shown.

Table S1. Sequence of sacbrood virus DNA used for dsRNA production

Virus	Sequence (5′→3′)	Target gene	NCBI reference
SBV	AGATGTGAACGCTTACCCTGATGAGCCTCGTACTACGTTGGACATAGCTC<br>GTATATGGGGCTTGAGGAGTACGTTTAATTGGGGATCAGGCGATGCGCAT<br>GGCAAAGAGTTATTTAATACCGTTTTGGACCCATGTTTGAGATTTTATGAC<br>CAGGATTACGAAGGACAAATAACCCCGATGGAATATGTAACTGGGTTGTA<br>TAACTTTTGGTCAGGGCCAATAGAGTTACGTTTTGATTTTGTTTCAAATGC<br>GTTTCACACTGGAACTGTGATTATATCAGCGGAGTATAATCGATCATCTAC<br>CAATACGGATGAGTGCCAATCTCACTCCACTTATACAAAAACGTTCCATTT<br>GGGAGAACAAAAATCCGTACATTTTACTGTGCCGTATATATATGATACCGT<br>GTTACGTAGAAATACGGCTAGTGCCTATTTACCGGTTACTGATAATGATAA<br>GGTAGATAATGTTAGTAAGGCGCAGGCTACGGGGATTAGAGCAGAGTCTA<br>AAATGAGAGTGAAAGTGAGAGTAGTTAATGTTTTAAGGCCTGTTGCCTCT<br>ACTACCTCAACTATAGAAGTTTTGGTGTATATGCGAGGAG	VP3, 596 bp	99% similar to Korean SBV; accession no.: HQ322114
